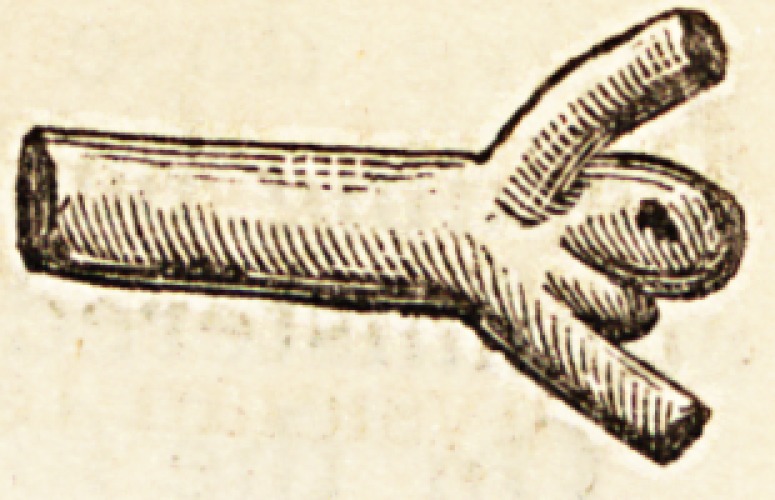# Remarks on Aneurisms of the Cerebral Arteries: With Cases

**Published:** 1835-01-01

**Authors:** Thomas King


*Remarks on Aneurisms of the Cerebral Arteries: with Cases.*
By THOMAS KING, M.R.C.S.

THERE are probably several distinct conditions of the parts within the skull, tending to the production of sanguineous apoplexy; and it has been long known, that miniature aneurisms of the cerebral arteries do occasionally, though rarely, give rise to fatal extravasations of blood in the brain. I do not, however, suppose that the bursting of such aneurisms is a very frequent source of apoplectic effusions; but the event is perhaps of much more common occurrence than it is considered to be. Under this opinion, I beg to introduce the following cases, by a few particulars that bear upon the subject of apoplexy generally.

Independently of the pulsating action of the heart, the more considerable arteries of the body are influenced by a gravitating column of blood, which, in ordinary circumstances, affects, in the greatest degree, the vessels of the most depending parts, and exerts less force on more elevated ones, in proportion as they are near the summit of the column. Now, the arteries, as well as the veins, are adapted to the precise quantity of distending pressure which each may have to resist, in the habitual attitudes of the animal: the most depending limbs possessing the thickest and firmest tubes; whilst the parts, which receive nourishment, in opposition to the power of gravitation, have vessels which are manifestly thinner and weaker; and the arteries, especially of the most elevated parts, are attenuated and incontractile.

Exceptions may be found to the preceding opinions, but they are chiefly such as the physiologist would account for in a manner corroborative of the principle just laid down.

The diminishing propulsive force of the circulation towards the vertex, in health, doubtless corresponds pretty accurately to the tenuity of the appropriate vessels, as well within as without the skull; but the prevalence of certain vascular diseases in the head—as nævus, aneurism by anastomosis, cerebral hernia, and apoplexy—strongly indicates that the adjustment between force and resistance is often destroyed.

The common atheromatous and ossific affections of the arteries occur in and out of the cranium, but not always in an equal degree in different parts of the same subject; nor do these particular senile changes appear to be necessarily connected with aneurism or sanguineous apoplexy.

After witnessing a number of apoplectic dissections, the above reflections seemed to invite a more close investigation of the extravasations; and, in consequence, out of eight or nine recent clots, examined with greater or less attention, three were found to afford small aneurisms. Two of these were evidently perforated; and two other cases presented very indistinct or doubtful traces of aneurismal dilatation.

In a post-mortem examination, after having incised the brain, with as little injury to the pia mater as the case will admit, the mode of procedure, to ascertain the state of the vessels, is to remove the whole of the pia mater, particularly that from the vicinity of the clot, about the base of the brain, and in the widest extent of the fissures of Sylvius. The membrane is then to be washed, perhaps macerated, and spread out under water, so that all the vessels may be distinctly traced.

It is, perhaps, to be anticipated, that the middle cerebral artery, and its subdivisions, will be most frequently the seat of dilatations; since this is the largest vessel from the circle of Willis, and it affords the greatest number of considerable branches distributed through the very deep and extensive sulci of the fissure of Sylvius, whence the greater extravasations are most commonly derived.

Aneurisms of the vertebral, basilar, or carotid arteries are in little danger of being overlooked; but the case is different with regard to morbid affections in the secondary vessels, whose course lies deeper in the interstices of the convolutions. It is to be remembered, that the tumours described are exceedingly small, rarely attaining the size of a pea; because the originally delicate vessel becomes still more reduced in forming the cell; and it is probably ruptured when the dilating cell is not larger than a large pin's head; and this, when empty and collapsed, would be almost imperceptible, unless anticipated and carefully sought for.

The following cases are abridged from much more detailed histories; and the illustrative parts are preserved in the Museum of Guy's Hospital.

The inquirer into this point of Pathological Anatomy will find some few literary references in Otto, under the head of Aneurism.

Feb. 12, 1831.—Henry N. æt. 45, a person of naturally delicate and otherwise enfeebled constitution, a married man, and not habituated to excess in drinking, had had several slight attacks of paralysis, to which were to be referred some loss of motion in one cheek, and an affection of speech. While under treatment for some considerable ulcers, attended with mercurial cachexia, he was suddenly carried off in an apoplectic seizure.

The dissection of the head took place before the body had become cold.

The depression of the convolutions, and compression of the intervening sulci, were very considerable over both hemispheres. A diffused ecchymosis, in parts amounting to a grumous stratum, occupied the cells of the pia mater pretty generally over the left hemisphere, but especially in its sulci. The lateral ventricles were distended with fluid serous blood, and the septum lucidum was destroyed.

One extensive cavity of grumous blood stretched through the substance of the left corpus striatum, into the central parts of all the three left cerebral lobes. This cavity was continuous with the superficial extravasation externally towards the fissure of Sylvius.

The coats of the right large cerebral artery seemed healthy, but within it were appearances of slow obstruction by coagulation. On the left side a second ramification of the great or middle cerebral artery presented a considerable ovoid tumour, which was situated in a sulcus. It had very thin coats, and was filled with a dark, solid, and stratified coagulum. It rather exceeded half an inch in length, and was somewhat less in width. The remainder of the artery seemed tolerably healthy; the ruptured aperture was not distinctly discovered, but this was doubtless the point whence the external and internal effusions had proceeded. Elsewhere the brain was natural, and no other traces were found to elucidate the primary attack of paralysis.

Aug. 6, 1833.—J. F. æt. 30, a stout bricklayer, presented himself in the evening, on foot, having been recently seized with severe pain in the head. He appeared much distressed, and almost stupefied; the pupils were rather contracted, and but very little acted on by light; the face uninjected; the pulse slow, but neither strong nor full; the tongue was white.

It was reported that previous to the attack he had been quite well, actively employed, and had been drinking very freely for several days. Presently after presenting himself, he became suddenly insensible; his arms were stretched by his side; his breathing was laborious and almost stertorous, and for a short time there was a little foaming at the mouth; the pulse was not much changed. He lost sixteen ounces of blood by cupping, and seemed materially relieved. He took a purgative dose of colocynth and calomel; and, at midnight, bleeding being deemed unadvisable, ten grs. of calomel were administered in addition.

On the following morning all the symptoms of apoplexy were developed in the highest degree. Eighteen ounces of blood were quickly abstracted from the temporal artery, and the pulse became much less hard and full; some convulsive action of the arms occurred. After the lapse of an hour (at 10 a.m.) v.s. was repeated to the amount of fourteen ounces, with a similar and marked impression on the pulse, but without any influence over the insensibility; the patient yawned: however, he died at 1 p.m.

The examination of the head only was performed 24 hours afterwards. The veins of the brain generally were moderately full, and the convolutions appeared a little flattened or depressed. There seemed exteriorly rather a deficiency of serous secretion, and the arachnoid membrane was partially clouded by diffused opake spots. A considerable lamina of black clotted blood occupied the cells of the pia mater, over all the central parts of the base, extending widely into the greater fissures, around the cerebellum, and, in some lateral sulci, even towards the vertex; and also accompanying the arteries of the corpus callosum. The substance of the brain in general was of a healthy firmness, and rather pale than otherwise. The lining membrane of the ventricles was unduly firm, and the third and fourth ventricles were actually granular.* All these cavities were much and permanently dilated, and they contained near 1½ oz. of serum, slightly imbued with the tint of port wine. Great part of the plain of the fornix, as well as its connexion with the corpus callosum, was in a state of apparent solution. A pretty solid clot of black blood was found distending the third and fourth ventricles, and continuing somewhat into the lateral ones, by the foramen of Monro, the circumference of which was partially dissolved. The floor of the third ventricle, anteriorly, was lacerated, softened, and dyed with ecchymosis, and a similar disorganization was found penetrating forwards into the substance of the right hemisphere, somewhat sinuously, and about 1½ inch in extent. The large arteries were all healthy in structure, with the exception of the right anterior cerebral, which offered, at its angle of bifurcation, a double aneurismal

* This appearance, which is not very uncommon, is probably a consequence of inflammatory action, which, in fact, had occurred in this case. A thin stratum of fluid blood, diffused over such granules, always renders them remarkably distinct.

swelling. In this, the proper mode of examination detected a rent, which was manifestly the source of the extravasation.

March 5, 1834.—Mary C. æt. 56, a tall, large, fat person, came under treatment four days after being seized with a severe apoplectic fit, which produced paralysis of the right limbs, and of the organs of speech. She had already been most actively depleted; and by the use of purges and some counter-irritants, &c., sensation and voice became nearly restored, and she could walk across the room with tolerable facility by the beginning of August.

Oct. 3d.—She suffered a second attack, by which the articulation was rendered very indistinct; her intellect became greatly oppressed, and the affected limbs much weaker. Cupping to the extent of xij. and viij. ounces was performed, with an interval of a week; small and repeated doses of calomel were administered, assisted by purgatives, and a blister.

Oct. 23d.—Under this treatment she appeared slowly mending, and thought herself much improved. She became restless early in the night. The next morning she was found insensible; and she died at 10 A.M. of the 24th.

The examination took place 26 hours afterwards.—The pia mater was greatly infiltrated with serum, and separated readily from the surface of the convolutions. The corpus callosum appeared scarcely at all arched. The vascular injection of the several parts of the brain was considerable, and the interior substance of the left hemisphere was extensively and materially altered, being much too firm, and of a dull reddish hue. Between the left thalamus and the lateral sulci was seen a plain cavity, nearly half an inch square, placed horizontally, and rather coloured than filled with dark ochrous matter. A recent apoplectic cell, about the size and figure of a small hen's egg, was found along the outer side of the left corpus striatum, and filled with a pretty uniform grumous solid. The cavity was lined by rugged ecchymosed medullary substance; and it had a small fissured communication with the lateral ventricle, above the striated body. The ventricle contained a good deal of blood-tinged serum, and some small spongy masses of coagulum. A small yellow soft cell was found in the inferior plane of the right corpus striatum. The arteries of the base were tortuous, rigid, and opaque; and a minute aneurism was detected, but apparently unbroken, in the sulci of the left fissura Sylvii immediately adjoining the floor of the great extravasation.

## Figures and Tables

**Figure f1:**